# Navigating identity: the experiences of Chinese women adopted into families

**DOI:** 10.3389/fsoc.2025.1615777

**Published:** 2025-07-07

**Authors:** Eve Neider, Xiafei Wang, Ting Guan

**Affiliations:** Syracuse Renée Crown University Honors Program, Syracuse University, Syracuse, NY, United States

**Keywords:** adoption, cultural identity, whitewash, racism, adoption stigma

## Abstract

**Introduction:**

This qualitative study explores the experiences of 12 Chinese women, aged 18–22, adopted by White families in the United States. While China’s one-child policy led to the international adoption of thousands of Chinese girls (1979–2015), qualitative research on their perspective about their adoption and cultural identity remains limited. Adoption is often misunderstood and stigmatized, particularly regarding its lasting impact. This study uses the intersectionality theoretical framework to understand the unique experiences of being Asian and adopted.

**Methods:**

12 participants took part in 60- to 90-min semi-structured interviews conducted in person or via Zoom. 17 questions explored topics such as feelings about adoption, identity, and experiences with racism. Narrative and thematic content analysis were used to interpret the data.

**Results:**

All participants expressed gratitude for being adopted but many felt embarrassed and uncomfortable discussing adoption, especially in childhood. Their environments shaped how they navigated identity—those in less diverse areas felt especially alienated. Many identified more with White culture than Asian culture. Most felt a stronger connection to White culture than to their Asian heritage and faced challenges being fully accepted by either White or culturally Asian peer groups. Every participant recounted instances of racism or being subjected to stereotypes.

**Discussion:**

Findings emphasize the importance of awareness and support from families, peers, and professionals. Social workers should consider adoptees’ cultural identity and emotional experiences in assessments and therapy. Educating adoptive families and partners on racial and cultural dynamics can reduce isolation and strengthen support for transracial adoptees.

## 1 Introduction

From 1979 to 2015, China’s one-child policy aimed to control overpopulation and boost the economy (Evans, 2000). The one-child policy was implemented with strict social control. Basically, families who exceeded the one-child limit were fined and lost jobs. Those unable to pay were denied a hukou, a legal document granting access to essential services such as healthcare, public schooling, and marriage registration (Zhao, 2016). However, the policy was more strictly enforced in urban areas, while rural families were sometimes allowed a second child if the first was a girl called a one-and-a-half child policy. For individuals living in rural areas, the process of birth registration was complicated and involved obtaining multiple documents and official stamps before a hukou could be formally issued. When applying for a hukou, parents who were unable to present a pregnancy certificate were required to pay a fine for having an “out-of-plan” birth (Kennedy and Shi, 2019). In 1982, most provinces implemented standardized rules for granting second-child permits, typically allowing exceptions for couples whose first child had a disability, those in remarriages where one partner was childless and the other had a child from a previous relationship, or couples who adopted a child after years of infertility but later conceived naturally (Scharping, 2003). The one-child policy contributed to gender imbalances and a small younger population, leaving fewer people to care for the elderly in China today (Hesketh et al., 2015) and substantial hidden human costs for Chinese people who live under this policy (Johnson, 2016).

Due to Confucian values prioritizing a patriarchal system, sons were preferred because they carried the family name and were expected to care for aging parents (Evans, 2000; Wang et al., 2020). Therefore, under the one-child policy, many girls have been relinquished in China, which contributed to international adoption of over 70,000 Chinese girls by American families (U.S. Department of State–Bureau of Consular Affairs, 2023). Between 1999 and 2022, 29.2% of foreign-born children adopted into U.S. families were from China (USAFacts, 2024). Those Chinese adoptees were mostly adopted by White middle-class American families, which position them uniquely at the intersection of being adopted, transnationalism, and a racial minority, raising important questions about their belonging, cultural connection, and psychosocial development (Shiao and Tuan, 2008). Understanding how this population navigates their identity will provide insights for child welfare scholars and practitioners.

### 1.1 Theoretical framework: intersectionality

Legal scholar Crenshaw (1989) introduced the concept of intersectionality to explain how systems of oppression and discrimination operate simultaneously across multiple social categories. Intersectionality provides a critical lens for understanding the unique positionalities of individuals whose identities span multiple marginalized groups (Crenshaw, 1989). For Chinese adoptees raised by White parents, intersectionality helps illuminate how adoptive, racial and cultural identities converge to shape their experiences of belonging and identity development. These adoptees may need to navigate complex terrains of cultural dislocation, such as bearing the loss and trauma of being abandoned, racial minorities in White spaces, and individuals adopted into families that may not share or fully understand their cultural heritage. Their lived experiences cannot be fully captured by examining race, adoption status, or family structure in isolation; instead, intersectionality demands that we consider how these identities interact within broader systems of power and privilege.

### 1.2 Being abandoned and loss of identity

Adoption, while often viewed as joyful for adoptive families, can be emotionally complex for children. Removed from familiar surroundings, children often experience sadness, anger, and fear. Early separation from birth mothers or foster caregivers can result in grief and feelings of abandonment. Learning that their biological parents could not raise them may impact a child’s self-esteem and sense of worth (Baxter et al., 2001). Because child abandonment was illegal in China, many families left infants in public places without leaving identifying information (Gillan, 2002). As a result, many adoptees grow up without basic information about their birthdays, birth families, or medical histories—essential elements of identity (Darnell et al., 2016). Many adoptees expressed a desire to find their biological families to complete their sense of self (Grigoropoulos, 2022). Most adoptees wondered about her biological ties and their history. For some individuals, adoption proved to be a challenging aspect of their identity, prompting the question “Who am I?” (Darnell et al., 2016).

### 1.3 Adoption stigma

Transracial adoption has held a disproportionately prominent place in the American cultural imagination, far exceeding its actual frequency. This topic seems to encapsulate many of society’s deepest concerns—such as the welfare of vulnerable children, ideas about love and race, and irresponsible parenting. Legal scholar Twila Perry insightfully notes that the debate over race and adoption reflects a broader conflict between two dominant perspectives in U.S. discourse: “race blind individualism” versus “color and community consciousness” (Briggs, 2012).

The concept of “adoptism” (Steinberg and Hall, 2000), is a form of microinvalidation that prioritizes the preservation of birth families while viewing adoptive families as less stable or emotionally bonded. Adoption stigma often frames adoptees as not being their parents’ “real” children. Adopted children may feel like secondary options when families are unable to have biological children. This perception can affect self-esteem and lead adoptive parents to feel inferior (Grigoropoulos, 2022). Simple questions like “Where is she from?” directed at transracial adoptive parents reflect societal expectations and values rooted in biological relatedness. These types of adoption microaggressions can also be internalized by adoptees. According to Baden (2016), describing adoption as a “win-win situation”—where the desires of childless couples and the needs of children for families are both fulfilled—can lead to a microfiction when deeper complexities, such as the birth parents’ relinquishment or the loss of personal history, are overlooked. In such cases, the real losses experienced by adoptees and adoptive parents are dismissed, and overly simplified, idealized narratives are created that ignore the emotional challenges, and nuanced realities of adoption (Baden, 2016). These experiences can create emotional distance and reinforce stereotypes. When people’s social or physical characteristics make others uncomfortable, Goffman (1963) suggests they are often driven to minimize their differences to appear as “normal” as possible.

### 1.4 Cultural identity: assimilation, intersectionality, and reculturation

In addition to the sense of being abandoned, loss of identity, and the stigma associated with adoption as experienced generally by all adoptees, Chinese children adopted into White families often face unique challenges related to cultural identity (Baxter et al., 2001).

The first salient factor for Chinese adoptees is the cultural disconnection. Most adopted Chinese children are placed with, upper-middle-class families. Although there is a persistent assumption that adoptees remain inherently connected to their birth cultures, adoptees grow up immersed in the White American culture of their adoptive families, which shapes their everyday experiences and identity more than their birth heritage (Baden, 2008). Children adopted internationally typically assimilate into their adoptive parents’ culture due to several key factors: (1) the necessity of communication for survival, (2) minimal or no exposure to their birth culture post-adoption, (3) the absence of cultural transmission from their birth culture, and (4) the psychological need to form secure attachments with their new caregivers (Baden et al., 2012). For example, up to 78% of Korean transracially adopted individuals (TRIAs) reported either believing they were White or wishing they were White during childhood (McGinnis et al., 2009).

The cultural disconnection is further complicated by racial differences. Transracially adopted individuals often begin to realize that their physical appearance differs from that of their adoptive parents around ages 4 to 5 (Lee et al., 2006). When a child first starts school, many activities are centered around traditional family structures, which may leave adoptees feeling different or like an outsider (Leever, 2023). Peers mocking their appearance is common (Clemetson, 2006). Racial microaggressions, such as microinsults and microinvalidations, which constantly challenge their legitimacy of family members in their adoptive families and hold them to expectations of ethnic authenticity (Baden, 2016). According to Lee (2003), transracial adoptees are frequently assumed to be “authentic” members of their racial or ethnic background, but they feel ill-equipped to meet these expectations due to their upbringing, and often feel compelled to disclose their adoptive status. Racial microaggressions further intersect with adoption microaggressions to shape adoptee’s identity development. Adoption microaggressions refer to frequent, often subtle remarks, insults, or actions that convey judgment, criticism, or bias related to adoption, such as suggesting adopted children are unwanted or not part of their family (Baden, 2016). Therefore, this intersectionality may aggravate transracial adoptees’ cultural limbo and heritage invalidation, suggesting their unique position between racial and cultural groups of not being accepted by both their birth and adoptive racial communities (Baden et al., 2012).

Because the intersectionality of racial and cultural factors forms their identity development, the Cultural-Racial Identity Model (Baden and Steward, 2000) highlights the importance of analyzing culture and race as distinct components in the experiences of transracial adoptees. They argue that exploring cultural identity and racial identity separately is essential for accurately capturing the complex and unique realities faced by transracial adoptees growing up in White families.

Finally, due to ethnic marginality and cultural displacement, transracially adopted individuals tend to assimilate into the adoptive culture such as fully embracing the dominant worldview, values, and beliefs when they were young (Chung et al., 2008). However, a process called reculturation often emerges in late adolescence or adulthood. Adoptees begin independently seeking connection with their birth culture (Baden, 2008; Shiao and Tuan, 2008). Reculturation becomes a coping strategy, helping adoptees process the complexities of racial identity and cultural belonging. Since adoption is a permanent and central aspect of their identity, adoptees’ attempts to reconnect with their birth culture are often deeply intertwined with the unresolved grief and identity negotiations stemming from their adoption experiences (Baden et al., 2012). According to McGinnis et al. (2009), though the majority of TRIAs assumed most White identity when they were young, most of these adoptees later felt a strong need to become more aware of their racial and ethnic identity, leading them to realign their self-identification with their Asian background.

### 1.5 Effects of parenting in the process of identity development

A key element in supporting adoptee identity development is open communication. Families that discuss adoption openly foster stronger identity development, which has been linked to higher self-esteem and fewer behavioral problems (Grigoropoulos, 2022). Darnell’s study (2016) also emphasizes early, open communication and how it can contribute to the adoptee feeling pride about their adoption.

However, despite many adoptive parents’ attentiveness to their child’s desire for belonging, they may unintentionally minimize the child’s need to explore a distinct personal identity. Fearing emotional distance, some parents may discourage this exploration, inadvertently hindering identity formation (Holtzman and Randolph, 2010). Although many adoptive parents today make efforts to expose their children to elements of their birth cultures—such as through dance, language classes, and other cultural activities—these experiences are often limited in depth and consistency. With few exceptions, transracially and internationally adopted individuals generally lack sustained and authentic engagement with the cultures of their birth countries (Baden et al., 2012). Further, research suggest that culturally competent parenting is crucial for helping adoptees navigate racism and developing psychological resilience (Mohanty and Newhill, 2006). U.S.-based studies show that ethnic socialization significantly influences the well-being of transracial adoptees (Duncan et al., 2021).

### 1.6 Current study

While the existing literature offers important insights into the challenges faced by transracial adoptees, several gaps persist. First, much of the research focuses on children and adolescents, with limited exploration of how adoptees make meaning of their experiences in early adulthood: a stage where identity exploration and reflection deepen. Second, most studies use quantitative approaches or aim to provide theoretical framework, leaving adoptees’ own narratives less heard. We need more qualitative work that centers adoptees’ voices, allowing them to articulate the complexities of adoption, culture, and identity. It is important to know how adoptees interpret and respond to racism, how they negotiate belonging within and outside their families, and how they imagine their futures in terms of parenting, and potential reconnection with their birth culture. By focusing on the perspectives of adoptees themselves, this study seeks to enrich the literature on transracial adoption and potentially inform adoption policy, parenting practices, and mental health support.

## 2 Methods

### 2.1 Data collection

Participants were recruited through snowball and convenience sampling. The first author reached out to Chinese adoptees that she knew and received referrals to additional participants through mutual friends. To avoid any bias with participants the first author knew, the first author specified the conversation was confidential and anonymous, used neutral language, and was transparent about their role as the researcher. Participants were contacted via text with a summary of the study, including its purpose, time commitment, anonymity, and an invitation to ask questions before agreeing to participate. The eligibility criterion was (1): adopted from China, (2): between 18- and 22-year-olds (this is the age most people are in college and are exploring their identities), (3): parents are White and, (4): identify as a woman. Eligible participants went through a 60- to-90-min interview consisting of 17 questions about their adoption and cultural identity. Interviews were conducted either in person or via Zoom. Before beginning, the first author reviewed the consent forms with participants and obtained either oral or written consent. To ensure participants felt prepared, they received the questions in advance. The research project was approved by the Institutional Review Board (IRB) at Syracuse University.

### 2.2 Data analysis

During the interview, participant’s answers were recorded, and the first author took notes during the interview and first impressions after the interview. The recordings were transcribed using Grain. The transcripts were then shared with the second author for review and co-analysis. The first and second authors read the transcripts of the 12 interviewees twice to familiarize themselves with the content. They later used a thematic content analysis to identify common themes and patterns in the participants’ responses (Braun and Clarke, 2006). For the initial few interviews, the first author coded the data by finding recurring words or phrases, which were then grouped into themes and used to develop a coding list in Excel. The second author reviewed the coding list, and together with the first author, reached a consensus to proceed with coding using the agreed-upon list. Particular attention was given to topics related to adoptees’ perception of adoption and ethnic identity. Additionally, there was an analysis of the frequency of recurring responses and answers to closed-ended questions. We constantly compared similar and different themes. For example, we found some participants’ parents intentionally connected them with Chinese heritage while some did not. Themes were then reviewed, refined, and assessed for uniqueness among participants. The thematic analysis was contextualized within existing research to explore possible meanings behind participants’ responses. The study also used narrative analysis to examine how participants constructed and conveyed their narrative (Riessman, 2008). The first and second author interpreted their responses, by analyzing their language, expression, and underlying feelings.

### 2.3 Trustworthiness

To enhance the rigor of data collection and analysis, we employed trustworthiness strategies as suggested by Lincoln and Guba (1985) and Padgett (2016). As experts in child welfare and qualitative research, the second and third authors reviewed the interview guides prior to the first interview to ensure dependability. For confirmability, we maintained a detailed audit trail documenting the entire data collection process. The first author kept field notes, memos, and self-reflections, and engaged in regular debriefings with the second and third authors throughout data collection and analysis to strengthen the credibility of the findings. To support transferability, we provided “thick description” by including verbatim quotes from participants to illustrate each theme and offer rich detail about the phenomenon under study.

## 3 Findings

### 3.1 Participant characteristics

At the time of the interview, all participants were 21 years old, except for one. Each had been left in a public place and later found by someone who brought them to an orphanage or foster care, where they lived until adoption. Most were adopted around the age of one, except for one participant with a disability who was adopted at age three. Most adoptive parents chose adoption due to infertility. Some parents specifically wanted a daughter after having biological sons. All participants believed they were placed for adoption because of China’s one-child policy. Some also speculated that their biological mother was too young or that their family faced financial hardship.

### 3.2 Perception of adoption

Given that adoption is a complex and profound life event that shapes every adoptee’s experience and sense of identity, we got deeper into understanding how adoptees themselves interpret this life-changing experience, which allows us to further explore how adoption influences their sense of self and identity.

#### 3.2.1 Overall positive perception of adoption

When asked, “How do you feel about being adopted, do you see it as something good or bad that happened?” Everyone said they saw it as something good that happened because they think they would not have had as many human rights or access to resources and opportunities in China. Some said they are glad they were adopted because they are grateful for their family.

*Participant 6: “I mean, I’m cool with it, I’m very thankful that I was. Obviously, it’s not perfect. There are issues, but overall, it’s a very good thing. I’m very privileged and thankful to be in a privileged environment.”*

*Participant 7: “I think it’s a good thing. I think the fact that my birth parents were able to say, okay, I don’t want to have this baby right now, so let’s put her up for adoption. I’m very grateful for that because I’m really grateful for the family I have here and the life that I have here.”*

#### 3.2.2 Negative perception of adoption as a child

However, many participants felt embarrassed about their adoption as children, as it set them apart and was difficult to fully understand.

*Participant 3 said: “I’ve made being adopted some big thing that everyone is going to react so terribly ever since I was so young and that’s not the case. I think now it’s just a sensitive topic. I remember when I was in middle school, my parents had friends over, and they said, like oh when we got her from China, and I remember I was so embarrassed… we used to celebrate my gotcha day, but I remember one year in high school, I was so embarrassed that they wanted to celebrate it and they just stopped after.”*

The feeling of isolation and being embarrassed about adoption during childhood could be due to a lack of shared experiences and understanding from others. It was not knowing other adopted children that made participants feel different, leading to discomfort and avoidance of the topic.

However, as they grew up, society became more informed and accepting, and they were more comfortable discussing their adoption.

*Participant 9: “It was just a sensitive topic. As a young kid, I didn’t know much about adoption except for what my parents told me and let alone I didn’t know any of my friends that were adopted at the time or anyone else. I felt kind of like I was the only one, so I felt like I didn’t want to talk about it because of that.”*

*Participant 12: “When I was younger, I used to think of it as something bad just because I never knew anyone else that was adopted, so I felt like the odd one out. I didn’t like to talk about it because I feel like nobody really understood or really knew anything about adoption. But now I’m comfortable talking about it because I feel like people are more educated and open to what it is. I didn’t like having to explain that I was different from everybody and what being adopted meant and just going into details about my life.”*

Some also expressed frustration over the uncertainty of their background, including their genetic history, knowing they might never have answers. This lack of information contributed to feelings of confusion and a sense of being lost in understanding their own identity.

*Participant 4: “I don’t think I fully understood what it meant to be adopted, like I always knew I was. I used to have these preconceived notions that adopted children, it’s because they were bad, or their parents didn’t want them for negative reasons. But as I’ve gotten older, I definitely think that it’s not something to be ashamed of and it happens. Especially being adopted was out of our control, but this was a policy and a rule, so our parents just had to abide by it… things that are just kind of a given to people that are biological to their parents, like what their parents look like, medical history, stuff like that, it just confused me when I was younger as to why I couldn’t have that like everyone else.”*

*Participant 10: “I was more sad back then that I will never probably know who my birth parents were. I think that bothered me more, like the fact that there’s a part of my story that I’ve never know.”*

#### 3.2.3 Experiences of openly sharing their adoption

Many participants responded that they do not mind discussing their adoption if someone asks, but they typically do not bring it up on their own. Over time, they have become more comfortable talking about it. Some adoptees mentioned that they have grown accustomed to people asking if they were adopted and the follow-up questions that often come with it. Others appreciated that their adoption makes them unique.

*Participant 1: “I don’t really care. It’s not like, oh I hate it, or, oh I love about it. It’s like, if you’re interested and you want to ask about it, you can, I don’t mind. So, I guess I lean partial towards, like yeah, I like talking about it and part of me is trying to hide it.”*

*Participant 4: “I try to make it really obvious to people that it’s not a negative thing that I’m adopted. It’s not something I’m ashamed of and I’m always happy to talk about my experiences because it’s very different than others and it’s very unique. I think talking about my experience is a way for me to also let people know about who I am and why I am the way I am and how it’s a part of my identity.”*

*Participant 5: “I guess when I was younger, I always thought it was weird or just different, so I didn’t like talking about it. I find it kind of hard to talk about it because all the kids are like do you have a dad? I’m like, no and they’re like, did he die? And I’m like no I’m adopted. But then it’s just so hard to explain to.”*

Two individuals use dark humor as a coping mechanism to navigate personal and cultural struggles, finding relief in joking about their experiences, even when the topics are sensitive or uncomfortable.

*Participant 1: “I don’t really think about it too much… Okay, maybe I do think about it a lot… I like to joke oh my mom used a hanger, so that’s why I came partially broken.”*

*Participant 2: “I love dark humor. I just like to joke about working in the rice fields and saying the Chinese slur to make people uncomfortable.”*

#### 3.2.4 Imagining the “what-ifs”

Participants were also probed about “what your life would have been like if you were not adopted?” Most believed they would have had fewer opportunities, lived in a low-income household, and experienced an unstable home life. While most had only imagined whether they had biological siblings, some also wondered about their biological parents. The majority expressed a desire to meet their biological family to gain insight into what their life might have been like, though they were hesitant about maintaining a long-term relationship. All participants said their adoptive parents were supportive of them seeking out their biological family, but some worried it might create awkwardness, stir up emotions, or make their adoptive parents feel sidelined.

*Participant 8: “I actually put my DNA into the family DNA systems, to find out if I had any siblings who were also adopted. So far, no luck, but I feel like I would want to know just to see what my life could have been, and would I have been better off with them, or would I have been happy with my life now that I have, since I am happy with it. So, it’s like a toss-up of, would I have wanted to live that life versus this life?”*

*Participant 11: “I feel like in China, there’s a lot of control over what people do there. I feel like we have a lot of freedom in the U.S. in just like, your job, occupation, and basically stuff that you feel the enjoyment out of instead of basically what they throw you into. I’ve always wondered if I’ve had siblings, but I haven’t really pictured what my parents would look like.”*

*Participant 12: “I don’t think I would because I feel like I was given up for a reason, and I don’t really feel the need to meet the people that gave me up because I’m content and happy with the life I have now that I don’t need the closure of why they gave me up or anything.”*

### 3.3 Cultural identity

Further, a significant challenge Chinese adoptees in the U.S. face is navigating their cultural identity, as they grow up in a society where they are a racial minority. Adding complexity to this issue, their family environments often do not reflect their cultural background, which is unlike non-adopted Asian American youth who are typically raised in culturally aligned households. In the following section, we specifically examined Chinese adoptees’ struggles with cultural identity and explored key factors that shape their cultural identity development.

#### 3.3.1 Feeling embarrassed

A few described being embarrassed if people knew they were adopted. They avoided showing pictures of their family or being around their family in public, so they did not have to explain how they were different. This effort might be because people try to minimize their differences when they feel uncomfortable about their social characteristics (Goffman, 1963).

*Participant 3: “Yeah, I’ll talk about my parents, whatever. But it’s like, the second they’re like, oh, can I see a picture of your mom? I’m like, oh, God, here it comes, which I hate because I shouldn’t be like ashamed, especially with someone that’s supposed to like me for me.”*

*Participant 5: “I would avoid the subject at all possible. My elementary school had this thing called lunch bunch where parents would come in and serve lunch to the kids, my mom did it once, and then I was like, oh, my God, she’s never coming back here. Like, that was so embarrassing. It is sad that we kind of had to be embarrassed about that.”*

*Participant 12: “It definitely made me anxious and nervous about if I ever had to show a picture of my family, because it was just very different from everybody else’s. I will say that I think being adopted presented more of a challenge for me in terms of telling people my life story, because I would have to tell people that I was, one adopted, two that my father passed away when I was really young, and three that my mom is now in a relationship with a same sex partner.”*

#### 3.3.2 Being misperceived and receiving wrong assumptions

Participants’ embarrassment and feeling uncomfortable was further reinforced by the potential misperception of their identities by others in public. Some mentioned they worry in public that people assume they are dating their White brother or that they are not part of the family because they do not look alike.

*Participant 3: “I think about it mostly, when we’re out in public, like if we’re ordering something, I always try to stand really close to my parents so everyone knows we’re together… if we’re all out to dinner people will either think like I’m my brother’s girlfriend or right away they’ll be like she was adopted. I definitely have identity crises because I’m like, what would I look like if I was their biological kid or what do my biological parents look like?”*

*Participant 4: “When I was younger, I was a lot more insecure about it. I think just because growing up in a predominantly White community and not looking like your parents, just really took a toll on being different than other kids and now I don’t care. Except the only thing is, when I do go out with my dad or my brother, I’m worried that people think we’re dating or married.”*

Participants were probed about their feelings when people assume their parents are also Asian. Most said words like “annoying, awkward, or sucks.” Only one participant has never had people assume one of her parents must be Asian and it does not affect her. Although it is frustrating, many said they understand why people assume this and cannot blame them.

*Participant 1: “I feel like it sucks in a way because nobody likes any assumptions about their lives. But I’m also like, we live in a world where we assume a ton. So, I understand you probably think or whatever, but I appreciate you ask.”*

*Participant 6: “It just makes me uncomfortable. If it was some random person, you don’t even know me. Like, why are you saying those things? Then with my parents, it makes me uncomfortable because you’re literally my parents, it’s very uncomfortable. It is more because you feel like you have to kind of explain, oh, I’m adopted. Then, a White person with a White kid doesn’t have to explain.”*

#### 3.3.3 Not proud of Chinese identity

The majority said they are not proud of and embrace their Chinese identity. They seemed upset that they felt that way and wished they did not. Some felt that this identity constantly reminds them that they are not their parents’ biological child, but they wish they could be.

*Participant 2: “I do kind of go back and forth between oh do I wish I took Chinese or was more cultured and what it’s like to be Chinese. Sometimes I feel pressure to live up to my parents’ expectations to make them proud. I guess since I’m not their technically biological child. I don’t really feel like I have to act or be a certain way now, which is nice, but I used to more when I was in high school because I was reminded of it more daily because most of the population in high school was White. I feel like I do wish sometimes that I were White or that I was my parents’ biological kid. So, I wouldn’t say proud. I would just say it’s something that I have learned to accept. Unfortunately, hopefully, it won’t be that way.”*

*Participant 3: “It makes me sad because if you just ask are you proud to be Chinese, I would say no. It makes me sad because I think part of the reason, I would say no is because I don’t feel Chinese other than the way I look. I think I would say no because growing up and even today it has caused me feeling isolated and feeling different, which is sad because I wish I could say yes. Why I’m not proud of it is because society has just painted Chinese individuals to be one way and when you’re not like that, it’s just like, no one understands anything. I feel like part of it is just because I’m not what people think Chinese individuals are supposed to be, like.”*

Some are glad their name does not sound Chinese and the ones that have a Chinese first or middle name are bothered by it.

*Participant 4: “When I was younger, I wanted to change my name to something that was less Chinese… So I think when I was younger, it was a lot harder to come to terms with my race and my race playing a part in my adoptive family, but now it doesn’t matter to me.”*

*Participant 7: “My middle name is Chinese, but my first and last name aren’t telling of my race. I always think that’s interesting, that I’m happy about that, even though my name probably just sounds White… I just feel like it’s different and I’m sure it’s just a projection, but I feel like people make assumptions based on that, and I don’t like when people do that.”*

Also, being Chinese or Asian, has affected their beauty standards and self-worth. For example, how people who are attracted to Asians are often teased they have an “Asian fetish.”

*Participant 2: “Like, definitely romance wise, it’s weird because no one goes for a person of color… no one would be like, oh my God, that Asian girl is so pretty or oh, yeah this popular guy has a crush on a person of color. So, that definitely sucked because I would always compare myself to literal people who did not look like me, and that’s what I was surrounded with. So that kind of affected me, mental health wise and thinking about my appearance and what’s pretty and what’s not and just being socially acceptable and stuff.”*

*Participant 3: “Yeah, I think it’s been hard because it’s either like oh the boy has an Asian fetish, and it’s like no, why can’t someone just like me for me? Or it’s like, oh the boy likes you because you look so different from other people or people will always be like, oh your kids are gonna look so different. I feel like people always have something to say about my race.”*

#### 3.3.4 Feeling White on the inside

To further display the cultural/identity disconnection, participants shared that they were mostly feeling White on the inside. Despite their parents’ efforts to incorporate Chinese culture into their upbringing, several adoptees resisted engaging with it during childhood because Chinese cultures were seen as foreign or irrelevant to their lived experiences. The adoptees have the internalized perceptions of being “basically White,” even when their phenotype visibly contradicted that.

*Participant 4: “I used to think that I was White because I didn’t have any Asian friends. Like, my parents tried really hard to incorporate my Chinese culture into my life, but I didn’t really want to because I didn’t want to be different than the other kids, and it just wasn’t something that I think I could fully comprehend.”*

*Participant 6: “Yeah, I definitely did when I was younger because I knew I was Asian, but I’d always be like, oh but I’m basically White. My Asian culture or whatever heritage didn’t matter because I was already in a White space. I never felt compelled to embrace it, I guess. I’ve always felt kind of in between the two. I’m obviously not White, but I’m also not Asian in the way of being raised in Asian culture. I was Asian, but I had no culture behind it… like, they would talk about their home life or whatever, and I didn’t really experience that.”*

*Participant 12: “I see myself more as a White person. But at the same time, I also think that obviously, it is important to talk about people being different and the differences that people do experience. I’ll talk about it when it needs to be talked about, and I’ll have serious conversations about that and everything. But I’m not gonna just further reiterate the fact that I am different.”*

On the other hand, the feelings of cultural disconnection and identity conflict as Asian adoptees were due to being raised in predominantly White environments. Adoptees were racially identified as Asian while lacking cultural knowledge or connection, often leading to feelings of exclusion from Asian communities. Adoptees used the words “coconut” and “banana” to reflect their self-concept. They faced embarrassment or judgment from both sides, such as not speaking the Chinese language or struggling with cultural practices, which intensified their internal conflict. It was challenging for smoothly building racial and cultural identity among Chinese adoptees when their upbringing did not include meaningful engagement with their heritage.

*Participant 1: “Like a coconut. I definitely feel it in a lot of my life. You want to know your culture and you want to be immersed and knowledgeable about it because people make so many assumptions about you, but it’s just like not how you were raised, not how life was given to you. My parents weren’t knowledgeable and teaching us that, they thought they were just giving us enough by taking us out of the orphanages that we were in.”*

*Participant 2: “I was not a stereotypical Asian person, so I also felt a little bit judged for that as well, because you don’t feel accepted by the White kids, but you also don’t feel accepted by the Chinese or Asian kids, because I didn’t really have much culture at all. I mean, my parents immersed me in a lot of Chinese culture when I was younger, which was cool, but it didn’t really stick with me through high school, and it wasn’t like visible to anyone.”*

*Participant 10: “Like a banana, yeah. Because I feel like growing up especially in college as I experienced more racism or stereotypes. I feel like, I’m not White enough for the White people. I feel like they view the world differently than obviously a person of color, but I feel like I’m not Asian enough for the Asian kids in my school. I’m kind of in this weird in between.”*

Some described being Whitewashed, which is having dissociated oneself from one’s ancestral culture by acquiring or attempting to acquire an American lifestyle. They felt guilty saying it but saw it as the reality.

*Participant 2: “My dad one time said I was a little banana because I was yellow on the outside and White on the inside, which is really funny to me. You know Whitewashed and stuff.”*

*Participant 10: “I definitely have been whitewashed. Some people will say that to me as a criticism, but I feel like I can’t really control the environment I grew up in.”*

*Participant 12: “Although I look Asian, I think I would act more White than I look, so you could say Whitewashed, I guess, but only to a certain extent.”*

#### 3.3.5 Confronting racism and stereotypes

All participants experienced some form of racism, stereotypes, or bias. Common Asian stereotypes and microaggressions they encountered included assumptions that they were good at math or spoke Mandarin. Examples of racist actions included peers pulling their eyes back and receiving Asian hate comments during COVID-19. These comments hurt participants’ feelings and reinforced a sense of being outsiders.

*Participant 4: “The first time I can remember someone being racist towards me was in first grade when a White kid pulled his eyes back… the first moment that I acknowledged and really realized that I’m different than other people, racially, and I have a different place because of that. But I’ve had a lot of microaggressions throughout high school of people getting me mixed up with the one other Asian girl in the class or my parents come in and they’re not expecting them to be White, just stuff like that.”*

*Participant 6: “You’d get the smaller comments, like the stereotypical things, like Asians can’t drive or they would pull their eyes back or whatever. But the things that they’re saying are, like, oh you’re good at math or you must be good at everything. It’s like even though those things sound good, they’re still harmful, you know?”*

*Participant 12: “People just start talking to me in mandarin or some sort of dialect in Chinese, or they’ll ask me rude questions like, do you want to eat cat or dog? There was one time I was sitting in the mall in the food court, and some random guy came up to me and asked me if I saw the new restaurant that I just opened up, and he pointed to the Chinese place.”*

Despite the “model minority” stereotype, which believes Asian Americans are inherently successful, only some said they feel like they must act or be a certain way because either they are Asian or have White parents. The ones that said yes, mostly said they feel like they must do well academically.

*Participant 1: “I don’t do anything that’s culturally Chinese, but I know I’m never going to have the same privileges in a sense. People are always going to see me differently and have a higher standard. I feel like us Chinese people have very high standards and if we don’t meet them, people always have their assumptions about us.”*

*Participant 4: “Yeah, I think when I was younger, and I think still now, I put pressure on myself to do well, at least academically, specifically, because I don’t want to be considered the dumb Asian.”*

#### 3.3.6 Family’s effect on adoptee’s cultural identity

Based on participants’ narratives, we found that parenting had a profound impact on adoptees’ cultural identity development. Specifically, the actions and approaches of their White adoptive parents significantly influenced how adoptees perceived their Chinese identity. For example, some parents made intentional efforts to expose their children to Chinese culture in hopes of empowering them to reconnect with their heritage and fostering a sense of identity affirmation.

*Participant 8: “I joined this group called Sisters of China, which was started by my childhood friend, which is basically girls of ages from like ten or something all the way up to like, 30, where we just kind of would meet like once or twice a month and talk about different things that were going on or the different holidays that were going on. So that kind of helped me learn more about the culture again.”*

For some parents, their efforts of connecting their children with their own Chinese culture were not always well-integrated into the adoptees’ lived experiences. In some cases, they backfired as highlighting the adoptees’ differences caused feelings of embarrassment or otherness. Two participant’s parents had Chinese students or au pairs live with their family to connect them to the culture.

*Participant 5: “I feel like that made our family stand out more in our town and I didn’t like that. If we had like a sports game the au pairs would pick us up and you would always get looks because their culture is so different, sometimes they would come in funny or different clothing, as a child, I was like, oh my God, this is so embarrassing.”*

Adoptees who expressed discomfort in discussing their identities often linked this to their parents’ cultural neglect or avoidance. The silence of White adoptive parents around issues of race and culture left many adoptees feeling uneasy about their cultural background.

*Participant 3: “I feel like they’ve kind of allowed it to be slept on, swept under the rugged. Now as I’m older, I still hate talking about it, and it’s kind of inevitable. I don’t blame them, but I wish when I was younger, they asked why it is something that you hate talking about.”*

In addition to the daily interaction with parents, how parents react to racism the adoptees experienced is also very salient to the adoptees’ identity development. There was a clear absence of proactive racial socialization. None of the adoptees recalled their parents preparing them for racism or bias as children, which left them feeling confused, unvalidated, and vulnerable during formative years. As a result, most participants said they hid their experiences with racism, bias, and stereotypes. Many believed that their White family, friends, or partners could not understand the racism Asians face or that they could sympathize but not fully grasp the experience.

*Participant 7: “I do think they understand. I think with our current political climate and what we’ve grown up in, I think they understand, with Trump being like the China virus, like that’s crazy, but I don’t think you can fully understand unless you’re the person.”*

*Participant 10: “My parents and especially my brother can’t fully empathize or understand how going through a PWI (predominately White institution) or mostly White elementary school and growing up in the mostly White area affected me and my image of myself growing up. I feel like they don’t fully understand because obviously they didn’t go through it. So sometimes they’ll be thinking I’m being overdramatic or something. That dramatically affected me and traumatized me that they’ve say that.”*

However, parents became more concerned about racism during COVID-19. When participants did share their experiences, some parents reacted with anger and frustration, while a few responded by insulting the perpetrator or advising their child to simply ignore it.

*Participant 2: “Not that I remember them sitting me down or whatever, but I feel like during COVID they were like people might be more hateful towards Asian people and you just have to be careful or just be like self-aware.”*

*Participant 4: “Usually when I tell my parents about these microaggressions, they get really upset and that they’re willing to do something if I want them to.”*

In some cases, adoptees not only lacked racial support from their adoptive parents but also faced direct or implicit racial hostility within the home. One participant’s parents have made racist comments toward her and insulted China, which has strained their relationship. At times, when she has conflicts with her parents, she has wished she had not been adopted.

*Participant 6: “But I feel like it’s kind of like that White savior complex like, I need to go save this child that’s suffering in China or whatever. I feel like they don’t really understand that I am a minority. I feel like they think I’m an exception or I’m not like that or whatever. They’re kind of insensitive to the fact that I am a minority. Me and my little sister will joke about things like you’re yellow, but then they’ll say it too and it’s not for them to say. Then also China’s a communist country or they always talk about Chinese people, like they’re ruining the country.”*

In some cases, parents’ alienation of their adoptees could be subtle.

*Participant 8: “She’s like, oh yeah, you’re smart. You’re smarter than all of us. I don’t think she means to say it like a stereotype or racism, but it sometimes can come off that way.”*

#### 3.3.7 The effect of social context on cultural identity

In addition to the influence of parenting, we also found that the racial and cultural demographics of the adoptees’ living environment, had a profound impact on their cultural identity. Growing up in predominantly White communities created a markedly different social context compared to living in more diverse settings. For most adoptees who lived in predominantly White communities, they lacked access to Asian traditions, language, and role models, which contributed to a weakened sense of cultural identity.

*Participant 3: “Predominantly White, and I think it’s affected my cultural identity because I’ve had no desire or I haven’t really been expected to learn the culture because there’s not a lot of it around me, which I think is really sad. I think growing up I just wanted to integrate myself and be like everyone else, but I think it’s because I’m from such a white town, I really didn’t have any exposure. The family that I babysit is a Chinese family and I’ve babysat them since high school and that has healed the younger part of me because I actually get to see a little bit of culture in a very authentic way.”*

Moreover, in these predominantly White environments, the dominant culture was positioned as the ideal, making many adoptees hesitant or even resistant to embracing their Asian identity.

*Participant 4: “I grew up in a predominantly White community, so I didn’t have any Asian role models or anyone to really look up to. I wasn’t wanting to accept my Asian identity because when you’re younger, you don’t want to be different from other people.”*

*Participant 10: “Mostly White people and I’d say it caused me to reject my cultural identity for a while. I definitely wanted to be White when I was younger, and it definitely made me more insecure, so I kind of had to work on that. Now I’m pretty secure in who I am and my race. But when you’re growing up, when all your friends are White and your family’s White, it’s like, well why can’t I be White?”*

In contrast, participants who grew up in diverse areas or small towns where everyone knew each other reported fewer struggles with their identity.

*Participant 7: “I think I always felt like I belonged because I’m from the city, I know that there are a bunch of different types of families and different types of people.”*

*Participant 11: “I feel like it was kind of cool in a way because I got to basically tell people about how I was adopted and everyone knows everybody in our small town, so it’s like not a big deal.”*

*Participant 12: “I think now that I go to a more diverse school, I know more people that are adopted, whereas the area I lived in from my childhood was very single raced, and it was really uncommon for there to be kids that were adopted. In Australia, I went to an international school, so I wasn’t the only person of color there so that definitely helped me become more accepting of my background and my childhood story and everything.”*

### 3.4 Reculturation

Most adoptees said they would visit China again. A few participants mentioned they would go only for vacation rather than to connect with their adoption or Chinese culture. Others expressed a desire to understand what their life might have been like and to learn more about Chinese culture.

*Participant 2: “I don’t think I would. I mean if it was like, oh, my parents want to go to China, for a trip, yeah… it’s never really appealed to me because that’s not really who I am, I guess, like I don’t really identify with. I think it would be more harmful than helpful in just having another identity crisis and wondering about my biological parents and wondering what life would be like if I wasn’t adopted.”*

*Participant 10: “Yeah, definitely. I mean, I want to see where I was born at one point because obviously, I forget everything. At least if I can’t see my birth parents, I want to see the country I was born in.”*

Participant 4 visited China for 2 weeks as a teenager and met her foster family but had no luck finding her biological parents. In college, she studied abroad in Hong Kong for 6 months which helped her come to terms with her adoption and identity.

*Participant 4: “I cried a lot because I think I like to say that my trip back to China was a big turning point for me in my identity and who I am as a person, because I think it filled a part of me that I didn’t fully understand without being in that country. The first day was dedicated to me meeting my foster family, and we went to their house and had dinner with them. It was like trying to make up for lost time. I finally started accepting my Asian identity. I think I did feel kind of isolated sometimes looking Chinese and then not speaking Cantonese and speaking English. I learned a lot about myself and living abroad and about being independent, and what it means to live in Asia.”*

### 3.5 Perceived self as a parent

Most adoptees said they want to get married and have kids in the future. Out of the adoptees that want kids, most said they would be open to adopting but want at least one biological child. The reasons they would adopt a child is because they can relate to the child, they want to give a child a better life, and there are too many children in foster care.

*Participant 5: “I think I maybe would have children of my own and then adopt a kid. I think it’d be cool to see a mix of me and the person that I love. I feel like if I were in a good financial place and could provide a child with a good life that I would adopt. I feel like I’m adopted, and I could connect with a child in that way.”*

*Participant 12: “I would, because there are a lot of children that are in orphanages or foster care, and they deserve to have a good life as well. I think it’s just since I was adopted, I just think it’s something that comes naturally.”*

Only a couple of participants said they want to get married but do not want children due to concerns about childbirth pain and financial responsibility. Participants 9 and 11 said they would consider adoption only if they were unable to have biological children. Some participants expressed hesitation about adopting because they did not want their child to experience the same feelings of not belonging that they did as adoptees.

*Participant 3: “I don’t think I would want to adopt a kid. And that makes me really sad because I want to adopt a kid and make someone happy, X, Y and Z. I think I wouldn’t want to adopt a kid because, I wouldn’t want them to feel how I felt. I wouldn’t want to feel helpless as a parent knowing that your child feels different from you no matter what you do.”*

*Participant 9: “I wouldn’t… it wasn’t like, my dream. I’d prefer to have my own kids, nothing against it, because obviously I’m adopted. I want them to be mine, like not saying if I would adopt them, they wouldn’t be mine, but you know what I mean.”*

## 4 Discussion and implications

Our study contributes to the research on transracial and transnational adoption by highlighting the voices of Chinese women adopted into White families. Using an intersectionality framework, our findings based on in-depth interviews show that adoptees’ lived experiences are shaped not only by their adoption status but also by their racialized and cultural positions in predominantly White environments. Our study also showed that the Asian hatred during the COVID-19 pandemic also changed the family dynamics for Chinese adoptees. Our findings illuminated the potential psychological and sociocultural tensions adoptees navigate, including the sense of loss, adoption stigma, cultural/identity disconnection, as well as the absence of racial socialization within family and community spaces. These findings provide important implications for child welfare policy makers and practitioners.

Consistent with the work conducted by Baden (2016) and Darnell et al. (2016), our study also revealed that adoption was perceived as a life-changing opportunity while it also introduced profound challenges in identity development. They particularly felt like an outsider being different than other non-adopted children. Participants in our study expressed gratitude for being adopted while simultaneously struggling with feelings of loss, confusion, and abandonment, especially when they were young. They might have struggled more as children because around 5 years old is when they begin recognizing racial differences and realizing that their physical appearance differs from their adoptive parents (Lee et al., 2006). It should be noted that in our interviews, many participants got emotional talking about their adoption and said it was their first time talking about it on a deep level. Those who claimed to be uncomfortable talking about their adoption often exhibited signs of unease during interviews, such as hesitation, brief responses, or tense body language. This suggests that adoption’s emotional impact is often overlooked, and people may avoid asking adoptees about their experiences for fear of making them uncomfortable. Holtzman and Randolph (2010) found some parents may avoid identity exploration fearing emotional distance from the adoptee. Adoptees reluctance to initiate conversations about adoption, and their discomfort when felt the need to do so, suggests an emotional trauma that is not fully processed within many adoptive families. Meanwhile, as research suggests, families engaging in open conversations about adoption will help adoptees develop positive views about their adoption and higher self-esteem (Darnell et al., 2016). There is a strong need for therapeutic spaces for adoptees and their families to process their grief and identity loss associated with adoption.

As the framework of intersectionality (Crenshaw, 1989) suggests, we further explored how adoptees’ racial and cultural positions shaped their identity development. Consistent with Baden’s (2008) work, our participants’ narratives presented pervasive cultural disconnection among Chinese adoptees. Adoptees are physically marked by their ethnicity but culturally alienated from it, such as being embarrassed about their Chinese heritage and anxious over being racialized as Asian. Instead, many of them internalized the cultural disconnect as identifying White inside, but in the reality, they are often perceived as Asian from the Asian community. The sense of neither fully being accepted in White nor Asian communities and feeling like a “banana” echoed with Baden’s work suggesting ethnic marginality (Baden et al., 2012; Lee, 2003). The adoptees have the internalized perceptions of being “basically White,” even when their phenotype visibly contradicted that (McGinnis et al., 2009).

Similarly, as Baden et al. (2012) suggested, some cultural engagements were not authentic and consistent for Chinese adoptees. Some participants described being Whitewashed and forced to assimilate into American culture because of their White parents. Baden et al. (2012) mentions how TRIAs naturally assimilate to adoptive parents’ culture because of minimal exposure to birth culture and psychological need to form secure attachments with their new caregivers. In our study, we also found some parents’ effort, such as hosting Chinese au pairs, backfired, exacerbating adoptees feelings of otherness and reinforcing differences. These findings suggest that authentic, culturally competent parenting is critical for adoptees identity development (Clemetson, 2006). Although many adoptive parents today make efforts to expose their children to elements of their birth cultures these experiences are often limited in depth and consistency making them feel isolated and embarrassed (Baden et al., 2012). Cohen (2007) noted that many adoptive parents have problems connecting their daughters to Chinese culture. Instead of superficially imposing cultural heritage on adoptees, a deep and open conversation discussing their adoption process, their insecurity and confusion in the adoptive families, as well as their coping mechanisms (e.g., dark humors) may be more helpful. Many adoptees explained they did not understand what being adopted meant as a child and do not like to mention their adoption, which could have been caused by a lack of discussion with parents and friends around their adoption. Darnell’s et al. (2016) study (2016) indicates that adoptive parents can support the development of self-esteem and pride about their adoption by maintaining open discussions about their adoption experiences.

Further, the framework of intersectionality (Crenshaw, 1989) is powerful to understand the sense of otherness expressed by most participants when we explored the racial microaggressions they experienced in their daily lives. Our findings showed that adoptees had to navigate both adopted- and race-related stigma and stereotypes. It was very unfortunate that many of them said their White parents could not fully understand their experiences of racial microaggressions (e.g., saying they are being overdramatic) or even impose harmful stereotypes or being racist themselves. Baden (2016) noted assuming things like an Asian individual with one White parent must be biracial are microaggressions which can be internalized by adoptees. Some are glad their name does not sound Chinese and being Asian has affected their beauty standards, which is largely due to racism and microaggressions that tease Asians for their ethnic names and physical traits. However, the pervasive Asian hatred in the COVID-19 pandemic increased parents’ awareness of racial discrimination their adoptive children encountered. Our findings revealed systemic gaps in how adoptive families prepare for and respond to racism that is universal in their adoptive children’s lives. It is crucial for parents to recognize their privilege as White individuals, acknowledge the racism their Asian child may face, and learn how to support them when it occurs. If the adoptee is embarrassed by their race or adoption status, this should be openly addressed in a safe space and maybe with a mental health professional. Research suggest that culturally competent parenting is crucial for helping adoptees navigate racism and develop psychological resilience (Mohanty and Newhill, 2006).

Interestingly, our findings on social context extends existing literature that mainly focuses on family impact on adoptees’ identity development. We found that adoptees raised in racially homogeneous communities often described rejecting or minimizing their Asian identity to fit in. Also, not knowing anyone else adopted made them feel isolated and different. In contrast, adoptees raised in more diverse environments or exposed to authentic cultural experiences, like living abroad, reported greater openness to exploring their heritage. This important finding suggests that the changes in our social context, especially an environment that espouses racial and cultural diversity, would be helpful for adoptees’ integrative identity development.

As we interviewed Chinese women in their late adolescence and early adulthood, we were able to get in-depth about the process of reculturation (Baden, 2008)—an emerging interest in reclaiming Chinese identity. Findings suggest adoptees who do not feel “White enough” begin wanting to learn more about their birth culture (Baden, 2008; Shiao and Tuan, 2008). While participants felt culturally disconnected, the majority expressed a desire to visit China or connect with their heritage, suggesting an evolving relationship with their birth culture. This finding aligns with Grigoropoulos’ (2022) findings that most adoptees want to meet their biological family to complete their sense of self. For instance, the participant who studied abroad in Hong Kong described this experience as transformative, offering emotional closure and enriching her understanding of identity. Most imagined what their life would have been like in China, which aligns with Darnell’s et al. (2016) findings that most adoptees wonder about their biological background. These narratives affirm reculturation as a vital coping strategy for healing from the trauma associated with transracial adoption. Further policies and programs of adoption may be considered including support for adoptees to connect with their birth cultures.

Finally, as research suggest that trauma may be transmitted through parenting (Wang, 2022), we found it would be important to understand adoptee’s identity development and their views on becoming an adoptive parent in the future. We found many participants expressed interest in adopting children in the future. Their willingness, even among those who found their own adoption difficult, signals a profound empathy and an aspiration to parent differently. In addition, the hope that they could have a child they can relate to also reflects loneliness and the sense of not being understood among those adoptees. However, several adoptees expressed hesitation about adoption, fearing their child might experience the same cultural dislocation and sense of otherness. These different perspectives highlight the need for adoption services that go beyond child placement to address the psychosocial complexities, even when those adoptees enter early adulthood.

## 5 Conclusion

We used the framework of intersectionality to examine the cultural and racial experiences and perceptions of adoption among Chinese adoptees, particularly girls. While much of the existing adoption research relies on quantitative methods, our study employs a qualitative, in-depth approach that offers a more nuanced understanding of participants’ lived experiences. While adoption granted many opportunities and socioeconomic privileges for those adoptees, being raised by White parents in the U.S. created challenges in identity development that are rooted in racialization, cultural disconnection, and adoption stigma. The participants’ narratives challenge simplified portrayals of adoption as solely beneficial and instead reveal an emotionally complex landscape.

Several limitations must be considered. We only interviewed 12 participants, and most were 21 years old which might not be representative of a larger population of Chinese adoptees such as adoptees from different generations and regions. Another limitation is the potential for confirmation bias, as the first author personally knows most of the participants. However, we used other strategies to ensure trustworthiness. Specifically, the first author collaborated closely with two faculty members, the second and third authors, who are experts in child welfare and qualitative research and had no prior relationship with any of the participants. The second author, who served as the thesis advisor, reviewed all interview transcripts and co-analyzed the data with the first author. The third author reviewed the manuscript at multiple stages and actively participated in critical discussions throughout the analytic process. We believe that this collaborative approach, involving multiple researchers with distinct perspectives and no personal connections to participants, helped mitigate potential bias and enhanced the trustworthiness of the study findings.

Our research offers important implications for supporting the healthy identity development of transracial adoptees. Adoptive families must engage in ongoing racial and cultural socialization that is authentic, consistent, and developmentally appropriate. Open conversations beginning early in life about what it means to be adopted, including discussions of grief, loss, and belonging, can help adoptees process their experiences and resist harmful stigmas or assumptions. It is also essential to validate and believe adoptees’ experiences with racism; while ensuring they have access to safe, supportive, and consequence-free avenues for reporting racial incidents. These insights underscore the need for judgment-free environments where adoptees feel safe discussing their adoption and racial identity. People in the adoptee’s social circle can support healthy identity development and challenge harmful biases by learning more about Asian cultures—through movies, books, music, and the history of Asian oppression—as well as by engaging in self-reflection on their own privilege and biases. Society must challenge harmful adoption narratives, such as the idea that adoptees are being “saved,” and instead reflect on underlying biases and privileges. By examining personal and societal assumptions, people can reduce the invalidation often experienced by Chinese adoptees who may not feel fully accepted by either their birth or adoptive racial communities. This validation is crucial to helping adoptees embrace both their Asian and American identities. Professionals in child welfare and mental health should be trained in adoption-competent and culturally responsive care. Broader societal and policy efforts must also focus on normalizing nontraditional family structures, dismantling model minority myths, and addressing adoption-related microaggressions. By understanding the nuanced intersectionality of being Asian and adopted we can empower transracial adoptees to build a secure and affirming sense of identity.

## Data Availability

The original contributions presented in the study are included in the article/Supplementary material, further inquiries can be directed to the corresponding author.
