# Supplementary material for: Navigating identity: the experiences of Chinese women adopted into families
Source: Front Sociol. 2025 Jul 7;10:1615777. doi: 10.3389/fsoc.2025.1615777 (PMC12277292; doi:10.3389/fsoc.2025.1615777)
Supplement: Supplementary file 1 [file Data_Sheet_1.pdf]

Interview guide

Thank you for agreeing to talk with me today about your experience being adopted from China and how it's affected your life.

This interview is part of my honors thesis. I would like to better understand the role Chinese adoption plays regarding an adoptees' cultural identity.

Before we get started, I want to let you know that:

- We appreciate your time and honest comments about this topic.
- You do not have to answer any questions that make you feel uncomfortable, and you can stop or even leave the call anytime you want.
- Is it OK if we record the interview? We will also be taking notes in case we miss something and need to go back to make sure we have captured your thoughts accurately.
- The information you provide will be summarized with all of the other responses for our report and you will not be personally identified.

Do you have any questions before we begin? Let's talk about your adoption.

1. How old are you and how old were you at adoption?
2. Do you know why you were put up for adoption and if not do you think of why you were? Why did your parents decide to adopt?
3. How do you feel about being adopted, do you see it as something good or bad that happened?
4. Do you ever imagine your life if you weren't adopted, what do you think that would've looked like?
5. Do you like to talk about it, if not why?
6. Would you want to meet your biological family if yes or no, why?
7. What has been your experience like being a different race than your parents and if applicable siblings?
8. Ethnically what kind of area did you grow up in and how has that affected your cultural identity?
9. What does it feel like when people assume things because you are Asian, like that one of your parents must be Asian?
10. Do you have a moment where you faced racism, bias, or stereotypes, what does that moment look like and what were your parents' responses?
11. Do you feel like your white family, friends, or partners understand the racism Asians can face?
12. How have your parents connected you to Chinese culture and is it something you are proud of/embrace?
13. Do you feel conflicted with your identity by being Asian, but growing up American with white parents and feeling "white on the inside"?
14. Do you feel like you must act or be a certain way because either you are Asian or have white parents?
15. Would you visit China and why?

16. Do you want to get married and have kids when you are older?
17. Would you adopt a kid and why?
